# ErbB4 as a Potential Molecular Target in the Treatment of Esophageal Squamous Cell Cancers

**DOI:** 10.1155/2014/124105

**Published:** 2014-11-04

**Authors:** Ke Zhao, Bao-Jun Chen, Zhi-Guo Chen

**Affiliations:** Department of Thoracic Surgery, The Central Hospital of Wuhan, Jiang'an District, Wuhan, Hubei 430014, China

## Abstract

ErbB4 is an important member of ErbB subfamily of tyrosine kinases receptor with overexpression in several tumors; however its biological role in esophageal cancer is poorly understood till date. The main objective of this study was to examine whether miRNA-140-5p could target and control ErbB4 expression at transcriptional level. The ErbB4 expressions in different cell lines were evaluated by western blotting and luciferase assay. Moreover, cell proliferation, apoptosis, and cell invasion studies were investigated using MTT, flow cytometry, and transwell assays. miRNA-140-5p remarkably downregulated the ErbB4 expression in EC9706 and TE-1A cell lines. Furthermore, miRNA-140-5p transfected cell significantly controlled the cell proliferation and enhanced the apoptosis of multiple cells. Additionally, miRNA-140-5p had marked effect on the DNA synthesis and caspase 3/7 activity in comparison to control cells. Specifically, miRNA-140-5p inhibited/repressed the cancer cell invasion and migration in a sign to have important biological role in esophageal carcinomas. Taken together, miRNA-140-5p could act as a potential molecular target in ErbB4 overexpressing ESCC cell lines paving the way for effective esophageal cancer treatment.

## 1. Introduction

Esophageal cancer is one of the most fatal cancers worldwide with high prevalence in East Asian countries (China) [1]. The overall 5-year survival rate for esophageal squamous cell carcinoma is extremely low (~20%) with high mortality rate [2, 3]. Present treatment strategies include chemotherapy, radiation therapy, and surgical removal of tumors; however, none can improve the tumor progression profile in esophageal cancer [4, 5]. In this regard, molecularly targeted therapies attracted significant attention of researchers in the recent past [6].

Studies have shown that ErbB/HER receptors which govern the multitude functions including the cell proliferation, cell differentiation, and signaling pathway mediated cell death in cancer cells [7, 8]. The ErbB gene includes four members: EGFR (ErbB1), ErbB2 (HER2/Neu), ErbB3 (HER3), and ErbB4 (HER4) [9]. Of this, ErbB1 and ErbB2 are one of the well-known oncogenes and many therapeutic moieties have been approved targeting these receptors. These tyrosine kinase receptors have been reportedly overexpressed in many cancerous tissues and considered as one of the important clinical markers [10, 11]. While ErbB3 finds limited clinical application in ESCC, recently, it has been shown that ErbB4 is upregulated in many tumor malignancies including esophageal carcinoma [12, 13]. Experimental studies showed that downregulation or targeting of ErbB4 suppresses the tumor progression to a great extent [14, 15]. Xu et al. demonstrated that extranuclear ErbB4 has considerable effect on ESCC [16] while Pang et al. showed that ErbB4 knockdown resulted in inhibition of ESCC migration and invasion activities [17]. In addition, in xenografts model, ErbB4 mediated apoptosis was shown to be essential for the antitumor effect of tamoxifen and cancer patients with ErbB4 expression responded very well with tamoxifen therapy [18]. Targeting ErbB4 results in the promotion of receptor dimerization and activation of various signaling cascades. The signaling pathways include mitogen-activated protein kinase (MAPK) and phosphoinositide 3-kinase (PI3-K)/Akt pathways [19, 20].

MicroRNAs (miRNAs) are small, endogenous, and noncoding RNAs of 20–22 nucleotides that suppress the translation/degradation and gene expression of messenger RNAs (mRNAs) at postblockade level [21, 22]. The miRNAs directly control the physiological processes such as cell proliferation, differentiation, division, apoptosis, and dysregulation of tumor cells that may have direct/indirect role on the pathological aspect of cancers by altering the important signaling pathways [23, 24]. Emerging results have shown that selective miRNAs are involved in cancer pathogenesis and can function as tumor suppressing agents/tumor suppressing genes or oncogenes according to the cellular context. Several groups have reported the clinical significance of miR-93, miR-141, miR-200, miR-214, miR-100, miR-143, and miR-145 in various cancers; however, the role of miR-140-5p in tumor growth inhibition is not reported [25, 26]. Specifically, miR-140-5p participation in the cell proliferation and cell apoptosis of ESCC cells has not been documented.

Based on these observations, clinical potential of miR-140-5p as a cancer biomarker or therapeutic agent has been studied in the present work. Therefore, the main aim of this study was to examine the expression of miR-140-5p in ESCC cell lines and to draw a linear relationship between ErbB4 level and miR-140-5p in the given cell line. We have studied the effect of miR-140-5p and anti-miR-140-5p on the apoptosis (BrdU incorporation and caspase 3/7) level on ESCC cells. Furthermore, we have carried out experiments to prove ErbB4 as a potential molecular/pharmacological target in the treatment of esophageal cancers.

## 2. Materials and Methods

### 2.1. Regents

miR-140-5p mimics and miRNA negative control mimics (miR-NC), miR-140-5p specific antisense inhibitor (anti-miR-140-5p), and its negative control inhibitor (anti-miR-NC) were obtained from GenePharma (Shanghai, China). The esophageal cell lines, namely, EC9706 and TE-1, were procured from Cell Bank of Shanghai (China). The cells were immediately cultured in RPMI media supplemented with 10% fetal bovine serum (FBS) and 1% penicillin/streptomycin in a controlled incubator.

### 2.2. Cell Transfections

ES9706 and TE cells were seeded in a 6-well plate at a seeding density of 1 × 10^6^ cells/well and allowed to attach overnight (15 h). The cells were then transfected with miR-140-5p and miR-NC (200 nm) until the cells reach 70–80% confluency. The cells were analyzed using Turbofect transfection reagent (Fermentas).

### 2.3. Luciferase Assay

3′UTRs of ErbB-4 were amplified using PCR from genomic DNA and conjugated into pMIR-140-5p-report (Ambion). Followed by, miR-140-5p target sequence was induced from site-specific mutagenesis kit (Stratagene). Sequencing was done to confirm all the constructs. ESCC 9706 cells were transfected with miR-140-5p and miR-NC mimics and incubated for 48 h. The luciferase activities were evaluated through Dual Luciferase Reporter Assay System (Promega) which was normalized by dividing firefly luciferase activity with that of Renilla luciferase activity.

### 2.4. Cell Proliferation Assay

The cell proliferation/cell viability was performed by means of MTT assay. Briefly, cells were seeded and incubated in a 96-well plate at a density of 1 × 10^4^ cells per well. The old media were removed and replaced with fresh media and the cells were incubated with pcDNA 6.2-GW/EmGFP-miR (mock) and anti-miR-inhibitors-negative control (control). And, miR-140-5p and anti-miR-140-5P were exposed to cancer cells and incubated for 24 h. The cells were washed and treated with MTT (5 mg/mL) solution, followed by absorbance that was measured by a microplate reader at a wavelength of 570 nm. The absorbance was measured using a sophisticated multidetection microplate reader (BMG LABTECH, Durham, NC, USA). All experiments were performed 8 times (8 wells).

### 2.5. Cell Invasion Assay

Cell invasion assay was performed using transwell chambers (Millipore, Billerica, USA) coated with matrigel. miR-140-5p or anti-miR-140-5p or mock transfected ESCC cells were trypsinized, washed, and resuspended in a serum-free RPMI 1640 medium. This suspension was added to the upper compartment of transwell chamber (with matrigel). Complete RPMI 1640 medium with 20% FBS was placed at the bottom part of matrigel chamber as a chemoattractant. The cells were allowed to incubate for 24 h after which noninvading cells were removed using a cotton swab. The number of cells invaded was fixed and stained with crystal violet (0.1%). The cells were counted using a light microscope.

### 2.6. Western Blot Analysis

Western blotting analysis was carried out by electrophoresing 25 *μ*g total proteins against 10% SDS-PAGE gel. This was transferred to PVDF membrane and incubated with primary antibody after blocking was performed with 5% skim milk for 2 h. The blots were incubated with horseradish peroxidase- (HRP-) conjugated secondary antibody for 2.5 h at room temperature. Then membranes were observed by exposing to X-ray film in dark followed by chemiluminescence reaction using ECL detection reagents (Amersham, Little Chalfont, Buckinghamshire, England) as per the instructions of manufacturer. Scion Image software was utilized for densitometry analysis.

### 2.7. Cell Apoptosis Study

EC9706 cells were seeded in a 6-well plate at a density of 3 × 10^5^ cells/well and incubated for 24 h. Cells were exposed to different formulations at an equivalent concentration of 1 *μ*g/mL for 18 h. Cells were harvested and resuspended with PBS containing FITC-labeled Annexin V/propidium iodide mixture. The analysis was performed using a flow cytometer (BD FACSAria II, BD Company, USA). Annexin V stains the cells in early stages of apoptosis (lower right square), while Annexin V/PI stains the cells in late apoptosis (upper right square).

### 2.8. BrdU Incorporation Assay

The cells were exposed to miR-140-5p and its corresponding mock (25 nm) mimics in a 96-well plate. The miRNA-transfected cells were trypsinized/harvested and incubated with 10 *μ*M of 5′-bromo-2-deoxyuridine (BrdU) for 3 h. The cells were fixed with cold ethanol/HCL and BrdU labelling/detection kit (Roche Diagnostics GmbH, Mannheim, Germany) was employed to determine BrdU as per the instruction of manufacturer's.

### 2.9. Caspase 3/7 Activation Assay

The cells were exposed to miR-140-5p and its corresponding mock (25 nm) mimics in a 96-well plate. The miRNA-transfected cells were trypsinized/harvested and used to measure caspase 3/7 activity using Caspase-Glo 3/7 assay (Promega) as per the manufacturer's instruction.

### 2.10. Statistical Analyses

*P* < 0.05 was considered statistically significant. The data are expressed as mean± standard deviation (SD) and performed in triplicate. Two-tailed, unpaired Student's *t*-test was used to calculate statistical difference/analysis using Microsoft excel or graph pad prism.

## 3. Results and Discussion

### 3.1. Expression of miRNA-140-5P in Surgical ESCC Samples

**Figure 1:**
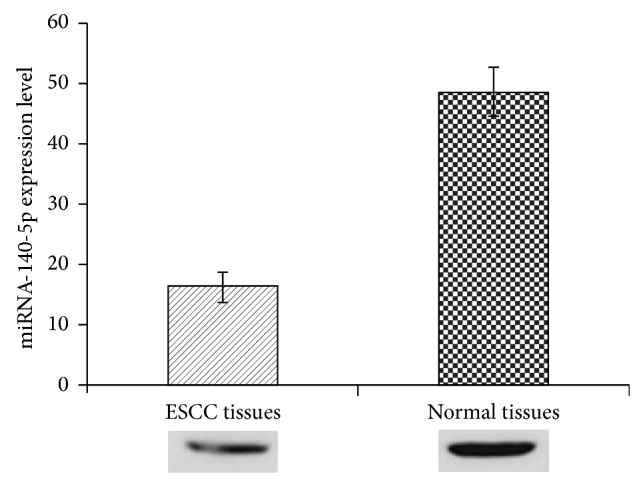
miR-140-5p expression in esophageal squamous cell carcinoma and matched adjacent normal cells. The expression of miR-140-5p in ESCC and normal cells was analyzed using immunoblot analysis.

To validate our postulation, miRNA-140-5P expression in ESCC surgical samples was investigated by qRT-PCR technique. As can be seen (Figure 1), miRNA-140-5P was significantly (*P* < 0.01) downregulated in ESCC cancer cell lines. In contrast, it is upregulated in adjacent normal cell lines where miRNA-140-5P plays a pivotal role.

### 3.2. Expression of ErbB4 in ESCC Cells

ErbB4 expression has been reported in multiple cancers including colon, ovarian, lung, breast, and esophageal cancers [27]. It has also been reported that ErbB4 has a great role in tumorigenesis and progression of cancer in clinical subjects. Moreover, it has been showed that somatic mutations of ErbB4 in metastatic melanoma result in carcinogenesis [28, 29]. However, reports of expression of ErbB4 in esophageal cancer cells are limited. Therefore, first we examined the ErbB4 protein expression in 4 different esophageal normal (HET-1A) and cancer cell lines (Eca109, Ec9706, and TE-1). It is evident from western blot analysis that all the esophageal cancer cell lines have expressed significantly higher levels of ErbB4 protein, while, on the other hand, HET-1A (normal cell) showed the lowest expression levels for ErbB4.

### 3.3. ErbB4 as a Potential Target for miRNA-140-5p

**Figure 2:**
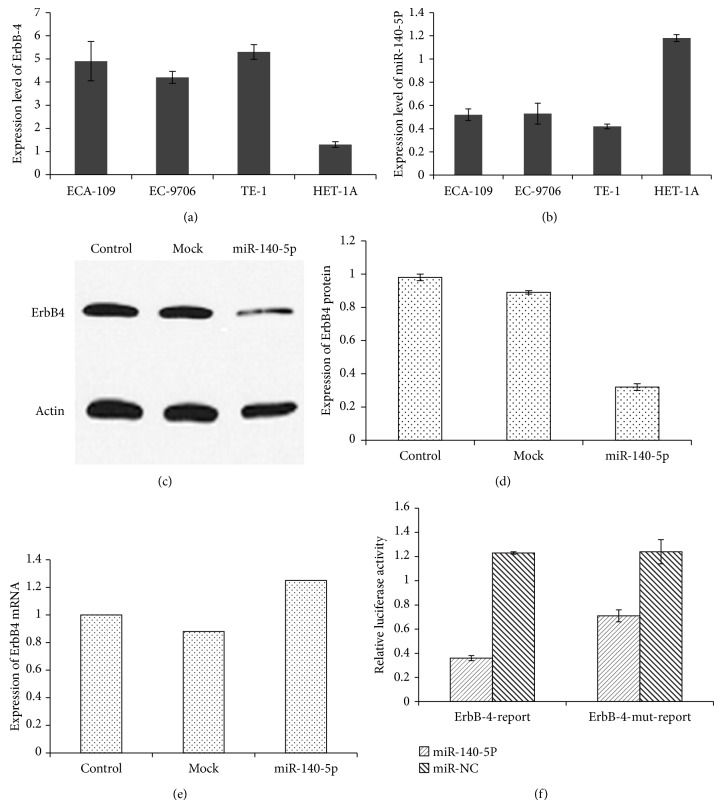
miR-140-5p controls the ErbB4 expression. ErbB4 expression in esophageal cancer cell lines (ECA 109, EC9706, and TE-1) and normal cell line (HET-1A) via western blot analysis (a). miR-140-5p expression in these cell lines (b). Western blot analysis of effect of miR-140-5p on ErbB4 protein expression (c and d). RT-PCR analysis of effect of miR-140-5p on ErbB4 mRNA expression (e). Analysis of luciferase reporter activity in EC9706 cancer cell line (f). The luciferase activity was examined by Renilla luciferase method.

Earlier, literature surveys have suggested that miRNAs play a central role in gene expression regulation and tumorigenesis. Studies especially suggested that miRNA expression is dysregulated in multiple cancers and miRNA may be a new targeting agent in the treatment of pathological cancers in the clinics [30]. Although miR-140-5p is involved in the tumor suppression, migration, and invasion properties, its therapeutic effect on esophageal cancer cell lines was not investigated and poorly understood until this date. Therefore, a linear relationship between ErbB4 and miR-140-5p was established by experimental parameters. It was observed that cancer cells (Eca109, Ec9706, and TE-1) with high expression of ErbB4 protein showed the lowest presence/expression of miR-140-5p while, on the other hand, noncancerous cell (HET-1A) showed maximum expression level of miR-140-5p (Figures 2(a) and 2(b)). Therefore, a negative or inverse relationship between ErbB4 and miR-140-5p has been established. Following this, we investigated the effect of miR-140-5p on the ErbB4 mRNA and ErbB4 proteins. Immunoblot analysis clearly showed that miR-140-5p remarkably decreased the expression of ErbB4 protein as seen by the lighter band comparing to that of thick and strong band in case of control cells (EC9706) (Figures 2(c) and 2(d)). However to our surprise, miR-140-5p did not have any effect on the mRNA expression (Figure 1(e)). This could be due to the posttranscriptional regulation of ErbB4 by miR-140-5p as expected. The results therefore showed that miR-140-5p remarkably decreases the expression of ErbB4 protein, while interestingly it has no or negligible effect on the ErbB4 gene expression. The results therefore clearly indicate the downregulation of miR-140-5p in cancer cells comparing to that of normal cells. This could explain the low or poor prognosis in esophageal cancer patients. Our in vitro analysis clearly showed that ErbB4 is negatively regulated with miR-140-5p wherein protein expression was limited upon treatment with the miRNA.

### 3.4. miRNA-140-5p Effect on Luciferase Activity

Next, we investigated whether ErbB4 is the pharmacological target of miR-140-5P or not. For this, luciferase reporter was constructed with 3′UTR of ErbB4 which was cloned with firefly luciferase gene. Now, EC-9706 cells were transfected with either miR-140-5P or miR-NC to examine the effect of miRNAs on the ErbB4 3′UTR with regard to relative luciferase activity. As can be seen (Figure 2(f)), miR-140-5P significantly inhibited the reporter with wild type ErbB4 3′UTR compared to that miR-NC which did not show any inhibitory effect while, on the other hand, analogous reporter with mutant ErbB4 3′UTR was relatively insensitive to miR-140-5P transfection. The observation suggests that ErbB4 3′UTR may contain the potential binding/interaction site for miR-140-5p [31]. The transfection showed that wild type ErbB4 3′UTR was prone to the inhibition of luciferase gene upon miR-140-5p exposure while that of mutation blocks the potential binding site in cells resulting in no inhibition of luciferase expression. This result clearly indicates that ErbB4 is a direct functional target of miR-140-5P in EC-9706 cell line.

### 3.5. miRNA-140-5p Controls the Cell Proliferation/Cell Apoptosis

**Figure 3:**
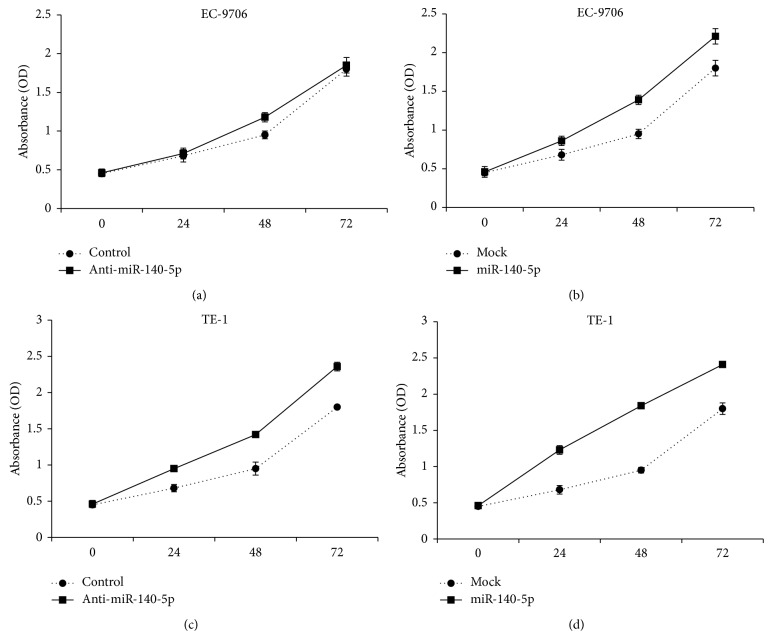
Effect of miR-140-5p on cell proliferation of esophageal cancer cells. Cell proliferation of EC9706 (a and b) and TE-1 (c and d) was analyzed after transfection with miR-140-5p and anti-miR-140-5p. The cells were exposed to respective mRNA sequence and incubated for 24, 48, and 72 h and growth inhibition was evaluated using the MTT assay.

**Figure 4:**
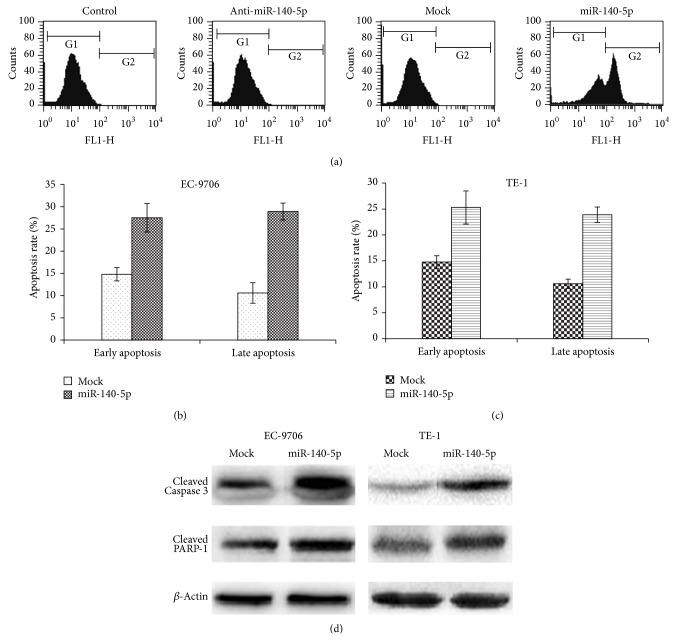
Analysis of effect of miR-140-5p on EC9706 cells via flow cytometer after staining with Annexin V (a). Percentage cell apoptosis was examined using flow cytometer after transfecting EC9706 and TE-1 cells with miR-140-5p (b and c). The percentage of cells in early and late apoptosis was quantified by interpretation of quadrant (UR and LR) with regard to control. The apoptosis assay was performed by staining Annexin V/PI on cells and analyzed by flow cytometry (d). The expression of apoptotic-related proteins, cleaved caspase 3, and cleaved PARP-1 was detected by western blot analysis, and *β*-actin was used as a loading control.

Having confirmed ErbB4 as the functional target for miR-140-5p, we next examined whether the miRNA can be effective to control or limit the cell proliferation activity in esophageal cancer cell lines such as EC-9706 and TE-1A cells. For this, EC-9706 and TE-1A cells were transfected with miR-140-5p and anti-miR-140-5p and cell viability was analyzed using the colorimetric MTT assay at 570 nm. As can be seen, cancer cells treated with anti-miR-140-5p remarkably increased the cell viability in comparison to the cells transfected with anti-miR-140-5p (Figures 3(a) and 3(b)). In contrast, cells transfected with miR-140-5p significantly decreased the viability or proliferation of both the cancer cell lines (Figures 3(c) and 3(d)). The results clearly suggest that miR-140-5p is acting as a tumor suppresser gene in the esophageal cancer cells and can potentially be a molecular target. In order to confirm whether inhibition in the cell proliferation resulted from the cell apoptosis, the transfected cells were treated with apoptosis kit (Annexin V and propidium iodide). Generally, Annexin has high affinity for membrane phospholipid phosphatidylserine that outlines the outer plasma membrane in the cell undergoing early stages of apoptosis (before losing its membrane integrity), whereas dead/damaged cells in the late apoptosis stages will interact with propidium iodide while viable cells with intact plasma membrane will reject it [32]. Therefore, FITC-Annexin V and propidium iodide combination will clearly identify the cells in different stages of apoptosis. In the present study therefore, transfected cells were studied by means of flow cytometry. Generally, control viable cells do not get stained with Annexin V and present largely in the G1 phase of cell cycle. However, cells get stained with Annexin dye when cells undergo apoptosis and therefore result in the shift of G1 to G2 phase of cell cycle. As can be seen, cells were predominantly present in G1 phase in case of control, anti-miR-140-5p, and mock group; however, in case of miR-140-5p transfected cells, G2 region was pronounced indicating that many of cells are in the apoptosis stage (Figure 4(a)). Furthermore, induction of apoptosis was quantified in terms of early and late apoptosis in EC9706 and TE-1 cell lines. As can be seen (Figures 4(b) and 4(c)), overexpression of miR-140-5p resulted in remarkable increase in the apoptosis rate comparing to the mock treated one. Importantly, we could find cells in both early and late apoptosis. Caspases play an important role in the progression of apoptosis and DNA fragmentation. Caspase 3 is especially capable of cleaving many cellular substrates including PARP. In the present study, cells transfected with miR-140-5p showed elevated levels of apoptotic markers, caspase 3, and PARP-1 indicating the cell death process (Figure 4(d)). The augmented enzymatic process was consistent with the flow cytometric analysis.

### 3.6. miRNA-140-5p Role in DNA Synthesis and Caspase 3/7 Activity

**Figure 5:**
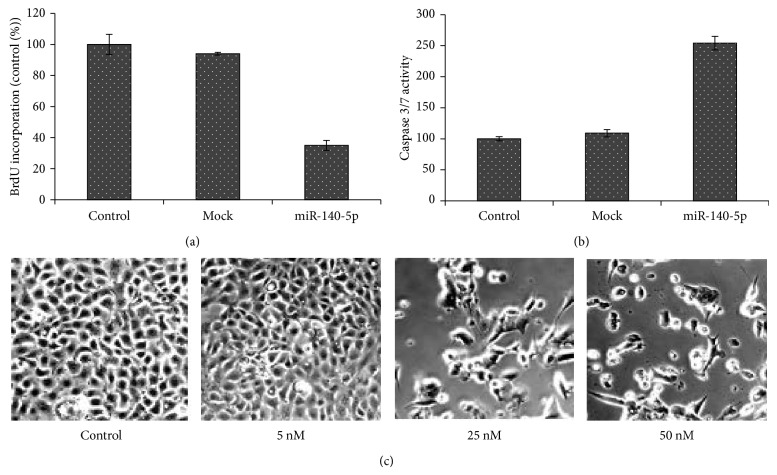
Antiproliferative effects of miR-140-5p on DNA synthesis (a) and caspase 3/7 activity (b). The miR-140-5p transfected cells were incubated with BrdU, followed by BrdU incorporation that was measured. Caspase 3/7 activity was measured as per the manufacturer's instruction. Values are expressed as mean ± SD from three independent experiments. Light microscopic photomicrographs of EC9706 cells after exposure to different concentration of miR-140-5p (c).

Consistent with the profound antiproliferative effect and apoptosis effect of miR-140-5p on esophageal cancer cells, its effect on the DNA synthesis to inhibit cell proliferation (EC-9706) was analyzed. To analyze DNA synthesis level, BrdU incorporation was examined. As can be clearly seen in Figure 5(a), miR-140-5p significantly decreased the BrdU incorporation in comparison to both control and mutated mocked cells that do not have the binding site for the miRNA. This indicates that the arrest in cell proliferation and apoptosis was resulted due to the DNA synthesis inhibition. This further suggests the influence of miR-140-5p in cell cycle resulting in the inhibition of DNA synthesis.

Flow cytometry analysis confirmed the role of miR-140-5p in apoptosis pathways. In order to further investigate its effect on the apoptotic pathways, caspase 3/7 activity was measured (Figure 5(b)). It was observed that nearly 2.5-fold increased caspase 3/7 activity was resulted upon transfection with miR-140-5p in the esophageal cancer cell. All these results point towards the fact that miR-140-5p has a significant role in the apoptotic cell death cycle in ES-9706 cell.

Apoptosis was further visualized by light microscopy wherein viable cells remained connected with specific morphology; however, for the addition of miR-140-5p, a slight change in morphology was observed (Figure 5(c)). The cellular morphology however markedly changed when the concentration of miR-140-5p was increased up to 50 nm. The cells were flat, round, circular, and distorted indicating the cellular stress and apoptosis.

### 3.7. miRNA-140-5p Effect in Cell Invasion and Migration

**Figure 6:**
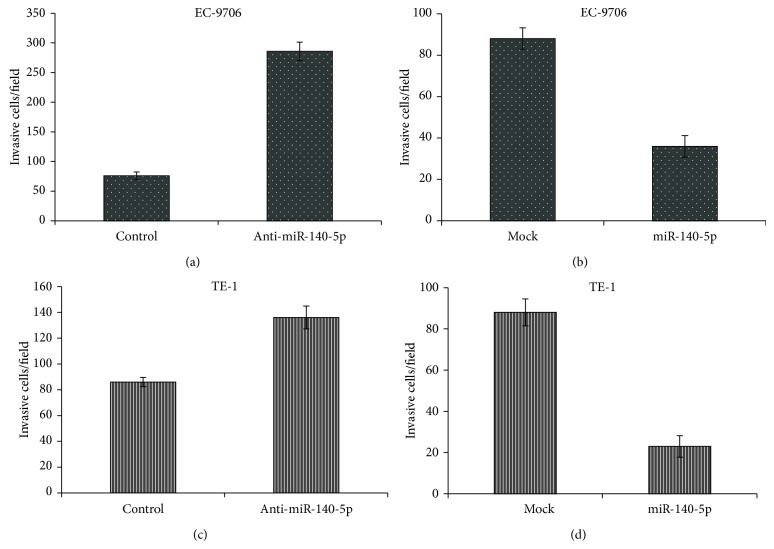
Effect of miR-140-5p on cell invasion of esophageal cancer cells. miR-140-5p and anti- miR-140-5p were transfected on EC9706 (a and b) and TE-1 (c and d) cells and cell invasion was analyzed using the transwell invasion assay.

Transwell assay was carried out to investigate the correlation between miR-140-5p and ESCC cell invasion. It can be clearly seen that overexpression of miR-140-5p significantly controlled the cell invasion in EC9706 cell lines (Figure 6(a)), while cell invasion was considerably high in which anti-miR-140-5p was transfected. This is in contrast with the downregulation of miR-140-5p gene expression. Similar trend was observed in TE-1A cells (Figure 6(b)) where miR-140-5p overexpression repressed the cell invasion. The study further reinforces the fact that the miR-140-5p controls the ErbB4 in ESCC cell lines by the multiple mechanisms including the cell proliferation inhibition, apoptosis induction, and cell invasion by downregulation and upregulation of expression level of miR-140-5p.

Consistent with cell invasion, migration of cells was examined in matrigel-coated polystyrene membrane. As expected, ectopic expression of miR-140-5p remarkably inhibited the migration of EC9706 cells by around 30–40% in comparison to control cells. Similar trend was observed for TE-1A cells which also showed remarkable inhibition of cell migration (data not shown).

Therefore, miR-140-5p has been shown to have a pronounced role in the tumorigenesis and progression of tumors. In the present study, we have showed that miR-140-5p remarkably downregulates the ErbB4 protein expression in both the esophageal carcinoma cell lines. Furthermore, our results suggest that miR-140-5p could directly target the ErbB4 receptor and could effectively prohibit the cell proliferation and repress cell invasion and cell migration of EC9706 and TE-1A cell lines. Therefore, overexpression of single strain of RNA can easily coordinate/regulate the gene expression on the cell related functions that will further encourage the therapeutic use of miRNA as a targeting agent. However, challenges in using therapeutic RNA are to precisely predict the molecular pathway or signaling pathway as a result of the binding interactions. In this regard, transcriptome analysis by microarray can be utilized to find out the miRNA target identification. The results therefore indicate that onco-miRNAs and suppressor-miRNAs could control the same gene with two different roles, either as oncogenes or as tumor suppressing genes depending on the targets.

## 4. Conclusion

Summing up, we have successfully showed that miRNA-140-5p could directly target ErbB4 protein in EC9706 and TE-1A esophageal cancer cell lines to control the cell proliferation and repress the cell invasion and migration. To the best of our knowledge, we for the first time demonstrated the correlation between ErbB4 and miRNA-140-5p in ESCC cell lines. For this first we examined the ErbB4 expression in normal and cancerous cell lines, followed by miRNA expression in these cell lines. Result suggested a significant downregulation of ErbB4 receptor in cancerous cell lines in comparison to normal cell lines. We have also demonstrated the cell proliferation regression in the miRNA-140-5p transfected cell in comparison to anti-miRNA-140-5p transfected cells. Finally we showed a remarkable apoptosis level, augmented DNA synthesis inhibition, and elevated caspase 3/7 activity with miRNA-140-5p transfected cell lines. The RNA transfected cells were observed to repress the cell invasive ability with parallel inhibition of migrations. Overall, our results suggest the potential application of miRNA-140-5p as a potential therapeutic moiety in the treatment of ErbB4 mediated/overexpressed esophageal cancers.
